# The important role of miR-1-3p in cancers

**DOI:** 10.1186/s12967-023-04649-8

**Published:** 2023-10-31

**Authors:** Shangming Dai, Fengjiao Li, Shuoguo Xu, Jinda Hu, Lichen Gao

**Affiliations:** 1 Department of Pharmacy, School of Pharmacy, Phase I Clinical Trial Centre, The Affiliated Changsha Central Hospital, Hengyang Medical School, University of South China, Changsha, China; 2 Hunan Provincial Key Laboratory of Tumor Microenvironment Responsive Drug Research, Hengyang, China

**Keywords:** miR-1-3p, Cancer, Mechanism, Chemotherapy sensitivity, Function, Targeted therapy

## Abstract

Cancer is a malignant tumor that seriously threatens human life and health. At present, the main treatment methods include surgical resection, chemotherapy, radiotherapy, and immunotherapy. However, the mechanism of tumor occurrence and development is complex, and it produces resistance to some traditional treatment methods, leading to treatment failure and a high mortality rate for patients. Therefore, exploring the molecular mechanisms of tumor occurrence, development, and drug resistance is a very important task. MiRNAs are a type of non-coding small RNA that regulate a series of biological effects by binding to the 3′-UTR of the target mRNA, degrading the mRNA, or inhibiting its translation. MiR-1-3p is an important member of them, which is abnormally expressed in various tumors and closely related to the occurrence and development of tumors. This article introduces miR-1-3p from multiple aspects, including its production and regulation, role in tumor occurrence and development, clinical significance, role in drug resistance, and approaches for targeting miR-1-3p. Intended to provide readers with a comprehensive understanding of the important role of miR-1-3p in tumors.

**Graphical Abstract:**

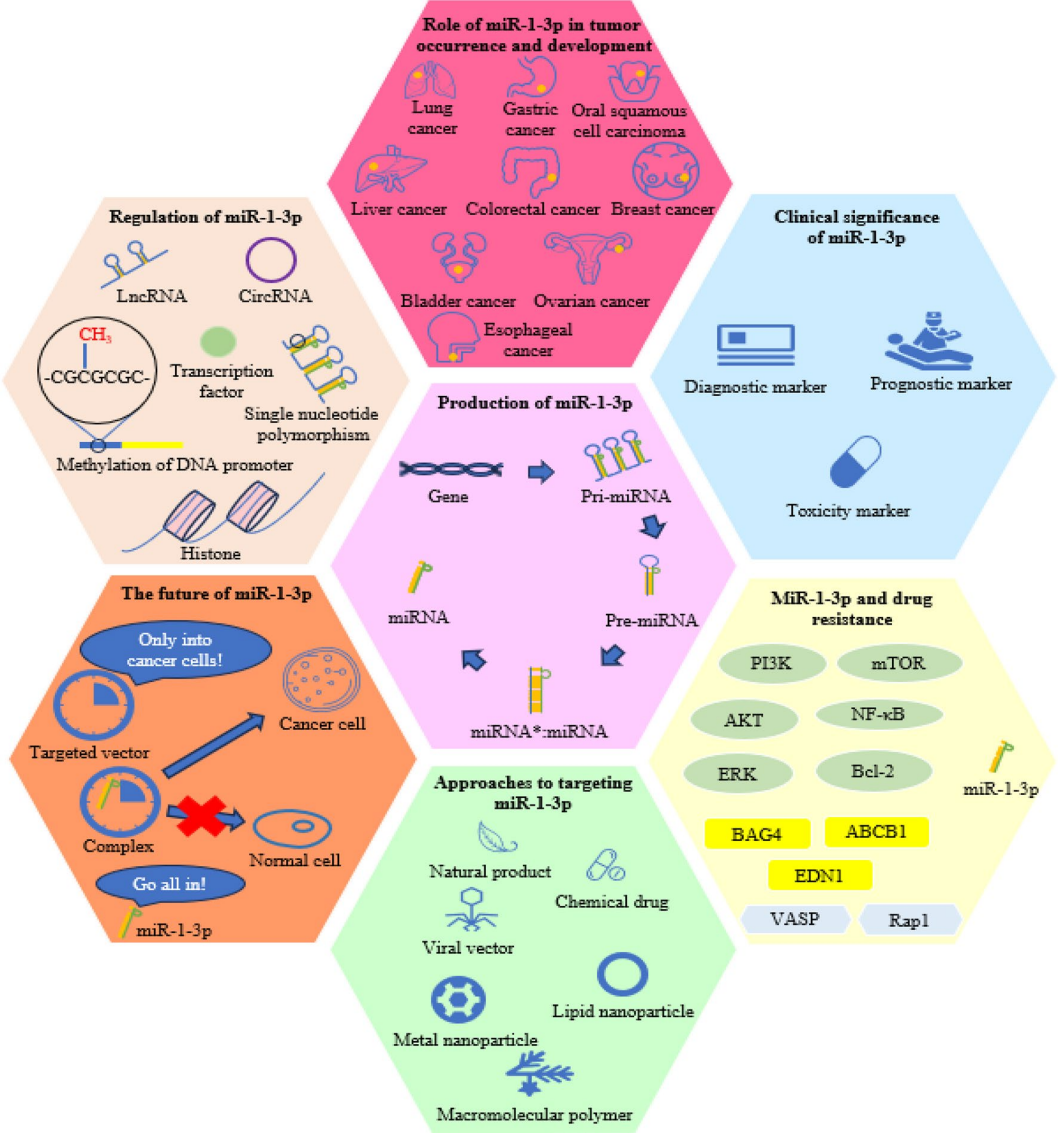

## Introduction

Cancer is a serious threat to human health worldwide. The latest cancer data reports that 1,958,310 new cancer cases and 609,820 cancer deaths are expected in the United States this year [1]. Meanwhile, China has also recently released its 2016 cancer data report. In 2016, it is estimated that there were about 4,064,000 new cases (crude incidence rate of 293.91/100,000) and 2,413,500 deaths (crude mortality rate of 174.55/100,000) in China [2]. The burden of cancer remains very severe. Currently, common cancer treatments include surgical removal, radiation therapy, and chemotherapy [3–6]. Surgical resection can directly remove the tumor site, but it may cause various postoperative complications, and some patients lose the opportunity for surgery when diagnosed with cancer. Although radiotherapy and chemotherapy have the ability to kill cancer cells, they have limitations and non-selectivity, respectively. More importantly, due to the complex mechanisms of tumor occurrence and development, drug resistance and recurrence often occur, leading to treatment failure and high mortality rates for patients [7, 8]. Therefore, it is very important to study the molecular mechanisms underlying the occurrence and development of tumors and the generation of therapeutic resistance. This may contribute to the development of molecularly targeted drugs.

MicroRNA (miRNA) is a class of non-coding RNA with a length of about 22 nt, which degrades mRNA or inhibits mRNA translation by binding to 3′-UTR of target mRNA, and regulates gene expression at the post-transcriptional level [9]. MiRNAs play an important role in maintaining normal cell metabolism. The abnormal expression of miRNAs may be related to the occurrence and development of various diseases, such as cardiovascular disease, neurodegenerative disease, cancer, diabetes, fibrotic disease, and inflammation. miR-1-3p is a very important member of the miRNA family, encoded by the miR-1–2 gene located on chromosome 18q11.2. Initial studies found that miR-1-3p was abundantly expressed in cardiac and skeletal muscle and is involved in their development [10–12]. miR-1-3p is able to directly regulate muscle differentiation regulators, including serum response factor, myogenic differentiation antigen (MyoD), and myocyte enhancer factor 2 (Mef2) [10]. Heart and neural crest derivatives-expressed transcript 2 (Hand2, a transcription factor that promotes ventricular cardiomyocyte expansion) has also been shown to be a target for miR-1-3p [10]. This miRNA also regulates myocardial physiological functions, and its aberrant expression has been associated with a variety of cardiac diseases, such as heart failure, myocardial infarction, cardiac hypertrophy, and arrhythmia. In recent years, miR-1-3p has been found to be highly conserved and consistently down-regulated in various tumors, and thus has attracted the attention of researchers. miR-1-3p is considered to be a tumor suppressor with great potential because of its ability to effectively inhibit a variety of tumors and improve the sensitivity of some anticancer drugs. In addition, miR-1-3p also plays a role in tumor diagnosis and prognosis. In the future, the function and mechanism of miR-1-3p still need to be further investigated, which will be beneficial to provide a solid theoretical foundation for clinical translation.

This article introduces miR-1-3p from various aspects, including the generation process and regulatory factors, its role in tumorigenesis and development, clinical significance, drug resistance, and targeted approaches.

## Production and regulation of miR-1-3p

### Production of miR-1-3p

**Fig. 1:**
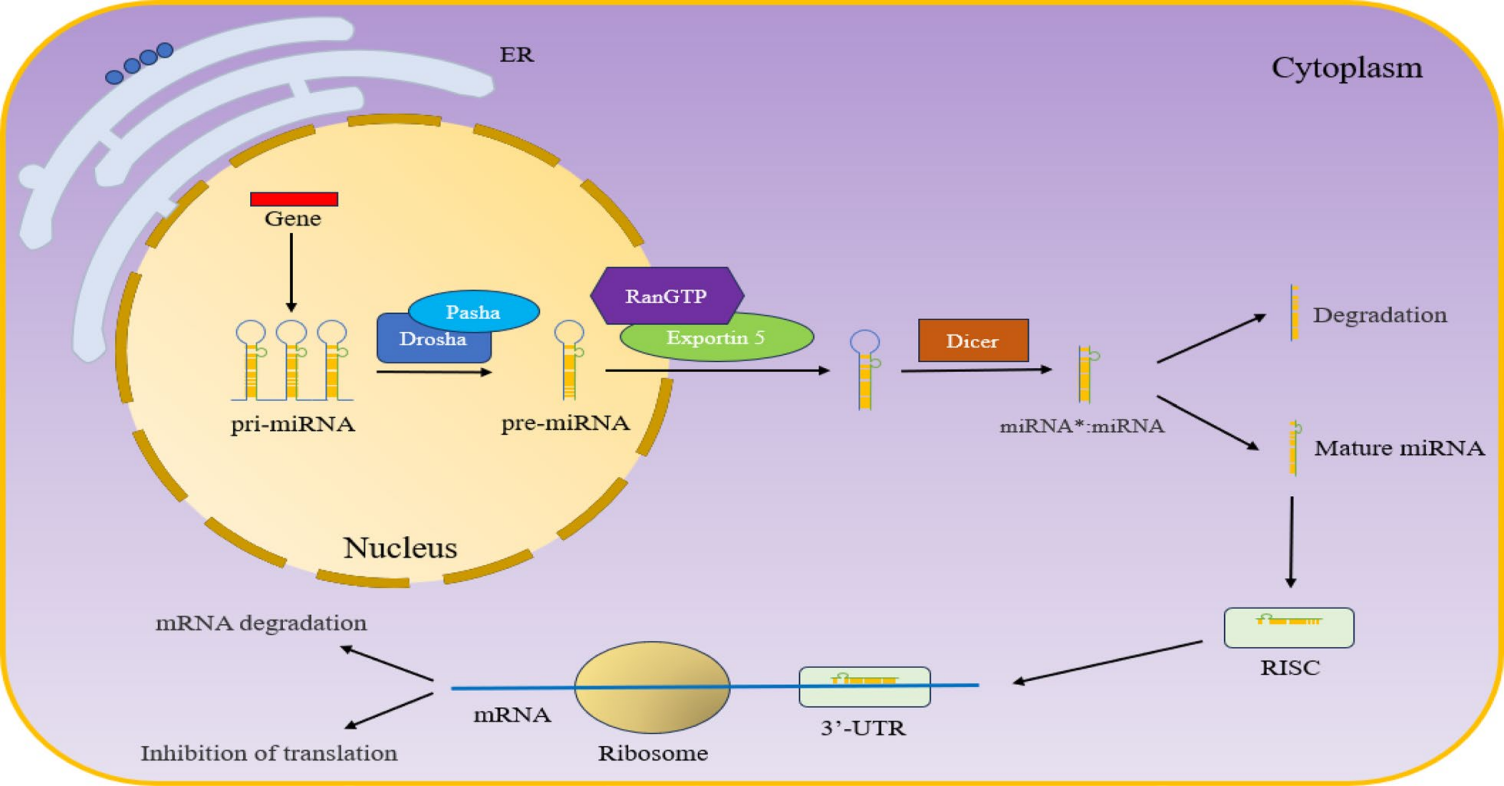
Process of miRNA production and processing. The gene encoding miRNA is transcribed into pri-miRNA, cleaved into pre-miRNA under the action of Drosha and Pasha, and then transported to the cytoplasm by RanGPT/exportin 5. Dicer in the cytoplasm further cleaves pre-miRNA into double-stranded miRNA (miRNA*:miRNA, miRNA* refers to a strand with very low or no expression). Subsequently, the double-stranded miRNA dissociates, with mature miRNA entering RISC and binding to the 3′-UTR of mRNA to degrade mRNA or inhibit translation

The gene encoding miR-1-3p is located in the intron region of the gene encoding protein MIB1 on chromosome 18q11.2 [13, 14]. First, the gene encoding miR-1-3p in the nucleus is transcribed into primary miRNA (pri-miRNA) under the action of RNA polymerase II [15, 16]. Under the action of Ribonuclease (RNase) Drosha and cofactor Pasha, pri-miRNA was cut into precursor miRNA (pre-miRNA) with hairpin structure, which was about 70 nt [17, 18]. Subsequently, pre-miRNA is transported from the nucleus to the cytoplasm through the RanGTP/exportin 5 transport mechanism [19]. The pre-miRNA in the cytoplasm is cut into double-stranded miRNA (combination of miRNA and miRNA*, miRNA* refers to a strand with very low or no expression) by another RNase III Dicer [20]. Afterward, miRNA and miRNA* are separated, where miRNA* is degraded, while mature miRNA enters the RNA-induced silencing complex (RISC) and binds to the 3′-UTR of the target mRNA, thereby degrading mRNA or inhibiting mRNA translation (Fig. 1) [21–23].

### Regulation of miR-1-3p

#### LncRNA

Long non-coding RNA (lncRNA) is a kind of non-coding RNA with a length of more than 200 nt. It can interact with DNA, RNA, and protein, thus participating in a series of biological processes [24]. As a competitive endogenous RNA (ceRNA), lncRNA can bind and silence corresponding miRNAs, thereby upregulating downstream mRNA and participating in a series of cellular biological processes. This is also one of the mechanisms that has been extensively studied. For example, lncRNA TUG1 can bind and silence miR-1-3p, thereby promoting the proliferation of liver cancer cells [25]. LncRNA MALAT1 can regulate migration and invasion in prostate cancer cells and survival and metastasis in esophageal cancer cells by targeting miR-1-3p [26, 27]. The exosomes secreted by breast cancer cells contain high expression levels of MALAT1, which can be transferred to surrounding breast cancer cells to silence the miR-1-3p in the cells and promote the metastasis of breast cancer cells and chemotherapy resistance [28]. In addition, RMRP, LINC00242, LINC01518, and DANCR have also been reported to silence miR-1-3p in non-small cell lung cancer, gastric cancer, esophageal squamous cell carcinoma, and glioma cells, respectively, promoting the malignant phenotype of tumor cells [29–32]. In summary, lncRNA is an important molecule that regulates miRNA levels within cells, and changes in its expression can cause changes in cellular function.

#### CircRNA

Circular RNA (circRNA) is a non-coding RNA that is covalently closed between the 3′ and 5′ ends, formed by reverse splicing through a special splicing method [33, 34]. Because circRNA is a circular structure with no polyadenylated tail at the 3′ end and no cap structure at the 5′ end, it is difficult to be degraded by nucleic acid exonuclease, so it is relatively stable in cells [35, 36]. The way circRNA regulates miRNA is similar to that of lncRNA, and it also acts as ceRNA binding to miRNA, thereby blocking the inhibition of miRNA on mRNA. For example, CircAGO2 can bind and silence miR-1-3p, thereby upregulating the expression of RBBP4. RBBP4 can deacetylate histones in the HSPB8 promoter region and inhibit HSPB8 transcription, thereby promoting the proliferation and invasion of colorectal cancer cells [37]. In addition, cHP1BP3 can also bind and silence miR-1-3p, upregulate the expression of C1GALT1, and promote the proliferation and migration of bladder cancer cells [38]. This indicates that changes in miRNA levels may also be caused by changes in circRNA expression.

#### Promoter DNA methylation

The dinucleotide structure formed by cytosine and guanine through phosphate linkage is called CpG. The DNA region rich in CpG is called the CpG island, typically between 200 and 1400 bp in length [39, 40]. CpG island is mainly located near the transcription start site of the gene promoter, which is an important occurrence area of DNA methylation [41]. High methylation at the CpG island site can lead to gene transcription silencing, while low methylation at the CpG island site promotes gene transcription [42–44]. Research has found that hypermethylation of the miR-1-2 gene (the gene coding miR-1-3p) promoter reduces the expression of miR-1-3p in prostate cancer. The decrease in miR-1-3p expression promotes the invasive ability of prostate cancer cells, which may be related to targeting downstream genes GOLPH3 and JUP [45]. Zhou et al. found that circSKA3 could increase the methylation of the miR-1 gene in glioblastoma, thereby reducing the expression of miR-1, and promoting the proliferation of glioblastoma cells [46]. During tumor development, high methylation of the CpG island of tumor suppressor genes is often observed. The methylation of the miR-1-3p gene leads to a decrease in its expression, which in turn promotes the occurrence and development of tumors, which is consistent. In summary, the expression of miR-1-3p is influenced by the methylation status of the coding gene, which is a regulatory factor worth paying close attention to.

#### Single nucleotide polymorphism

Single nucleotide polymorphism (SNP) is a common heritable variation, which refers to the DNA sequence polymorphism caused by the variation of a single nucleotide in a gene. SNP can be caused by the conversion or reversal of individual bases, as well as the insertion or deletion of bases, with single base conversion being the most common. Li et al. found that the serum miR-1-3p expression level in patients with abdominal aortic aneurysm (AAA) of rs2155975 AG + GG or rs4591246 AG + AA genotype (two SNPs located in pri-miR-1-3p) was significantly reduced, which was related to postoperative all-cause mortality and overall survival rate [47]. In addition, it was found that the SNP rs4591246 in pri-miR-1-3p also downregulated the expression of mature miR-1-3p in abdominal aortic aneurysm tissue, and then promoted the transformation of cell phenotype by upregulating TLR4, which was closely related to the risk of AAA patients [48].

#### Other ways of regulation

**Fig. 2:**
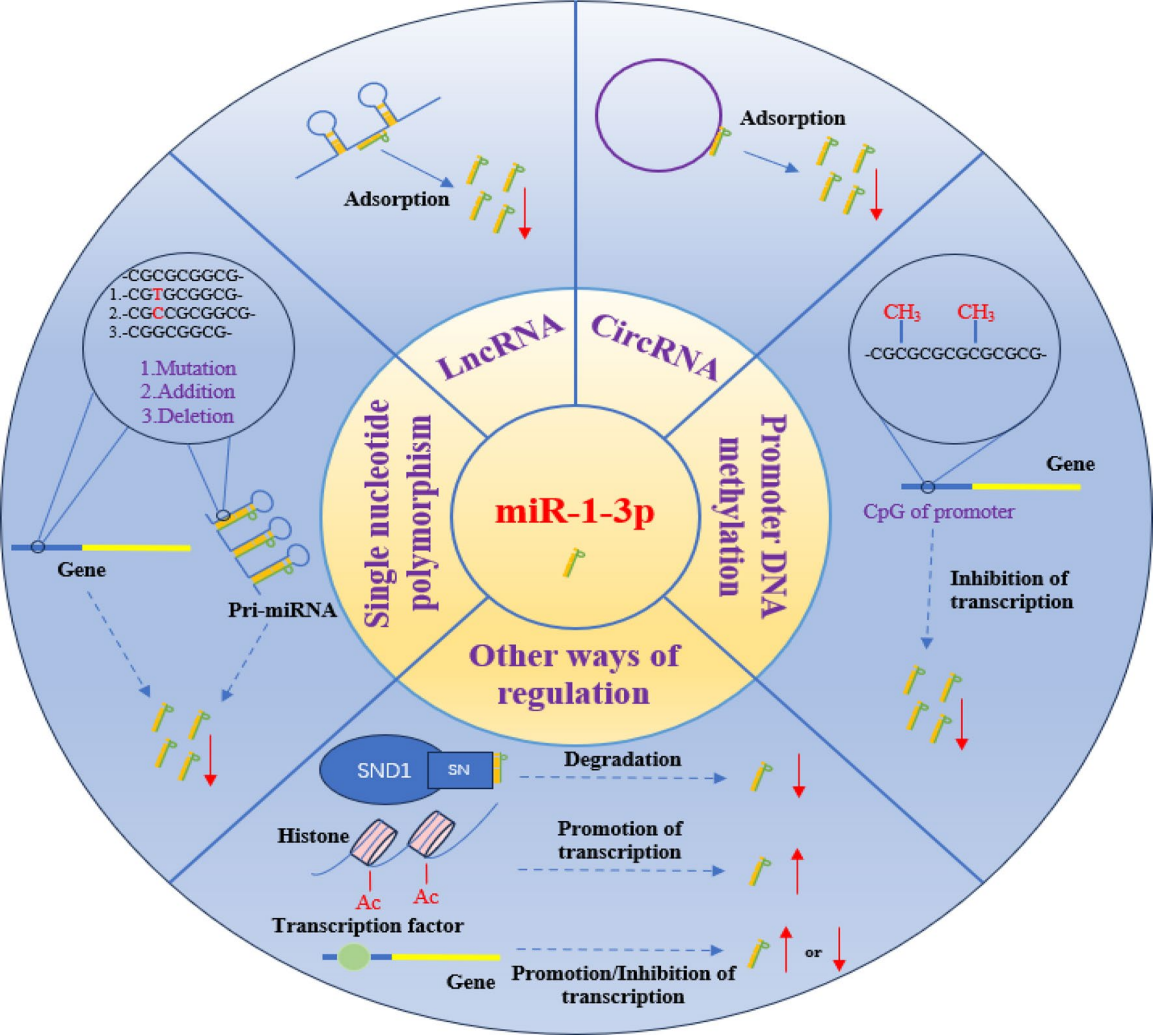
Regulation of miR-1-3p. The regulation of miR-1-3p is mainly influenced by factors such as lncRNA, circRNA, DNA promoter methylation, SNP, etc. LncRNA and circRNA can bind and silence miR-1-3p. DNA promoter methylation can inhibit the transcription of the miR-1-3p gene. SNP can affect the binding of related enzymes, thereby affecting the generation and processing of miR-1-3p. SND1 can bind and degrade miR-1-3p through the SN domain. In addition, factors such as transcription factors, histone acetylation, and gene mutations also regulate the expression level of miR-1-3p

SND1 (Staphylococcal Nuclease and Tudor Domain Containing 1) is an RNA binding protein, which is reported to play the role of nuclease in RISC, and also has the function of degrading hyperedited pre-miRNA and mediating the degradation of a group of mature miRNAs [49–52]. Recent reports have shown that SND1 can bind and degrade specific miRNAs through the SN domain, and its activity is related to the template [51]. The inhibition of SND1 can increase the expression level of miR-1-3p in colon cancer cells and enhance the sensitivity of tumor cells to the Bcl-2 family inhibitor navitoclax [52]. In addition, acetylation of histones, various mutations in coding genes, and changes in transcription factors may all affect the levels of miR-1-3p (Fig. 2).

## Role of miR-1-3p in tumor occurrence and development

### Gastric cancer

Gastric cancer (GC) is a common tumor of the digestive tract. The incidence rate of gastric cancer ranks fifth among all kinds of tumors, and the mortality rate ranks fourth [53–55].

Research has found that miR-1-3p was low expressed in gastric cancer tissues and cells, and was closely related to the size of the tumor. Overexpression of miR-1-3p inhibits the proliferation and invasion of gastric cancer cells by targeting stanniocalcin 2 (STC2) or centromere protein F (CENPF), in which CENPF is also associated with migration [56, 57]. Interestingly, miR-1-3p can target glucose-6-phosphate dehydrogenase (G6PD) to affect the Warburg effect (aerobic glycolysis) of gastric cancer cells. It can reduce glucose uptake, lactate production, and ATP production, inhibit cell proliferation, and promote cell apoptosis [30]. G6PD is a key rate-limiting enzyme in the pentose phosphate pathway (PPP), and how it participates in the regulation of the Warburg effect still needs further study [58]. It is worth noting that the G6PD-mediated PPP pathway is the main way to generate NADPH, which is the common reduction equivalent in the four major defense systems of ferroptosis (GPX4/GSH, FSP1/CoQH_2_, GCH1/BH4, and DHODH/CoQH_2_) [59–62]. The lack of NADPH can lead to ferroptosis, which is considered as a biomarker of ferroptosis sensitivity. However, it has also been reported that excessive NADPH can generate reactive oxygen species (ROS) under the action of NADPH oxidase (NOX), thus promoting the occurrence of ferroptosis [63]. Therefore, the role of NADPH in ferroptosis has a dual role, which needs specific analysis in different situations.

### Colorectal cancer

MiR-1-3p exhibits low expression in colorectal cancer tissues and cells. In primary colorectal cancer, the expression of miR-1-3p is closely related to tumor grade and overall survival in CRC patients [64]. MiR-1-3p can significantly inhibit the proliferation and invasion of CRC cells, which is related to targeting tyrosine 3/tryptophan 5 monooxygenase activation protein zeta (YWHAZ). YWHAZ can promote the epithelial mesenchymal transition (EMT) process in CRC cells, increasing β-catenin and N-cadherin, while reducing the expression of E-cadherin [65]. Ye et al. found that propofol could inhibit the proliferation of CRC cells and promote their apoptosis. The mechanism is that propofol can upregulate the expression level of miR-1-3p in CRC cells, thereby targeting insulin-like growth factor 1** (**IGF1) and inhibiting the activation of the AKT/mTOR signaling axis [66]. In previous studies, it has also been shown that IGF1 can bind to insulin-like growth factor 1 receptor** (**IGF1R), promoting the activation of the AKT/mTOR signaling pathway, thereby affecting cell proliferation, apoptosis, and metastasis [67–69]. Lv et al. found that nicotinamide phosphoribosyl transferase (NAMPT) was highly expressed in tumor tissues of CRC patients and is closely related to invasion, TNM staging, and low overall survival rate. NAMPT can activate transforming growth factor-β (TGF-β) signal pathways (upregulation of Smad 2, Smad 3, Smad 4, p-Smad 2, p-Smad 3 levels) promote the secretion of transforming growth factor-β1 (TGF-β1). And TGF-β1 can upregulate the level of miR-1-3p, which targets and silences NAMPT, ultimately forming a negative feedback pathway [70]. Targeting miR-1-3p to intervene in the negative feedback pathway for the treatment of CRC may also be a novel perspective.

### Lung cancer

Lung cancer is the cancer with the highest mortality rate worldwide. Lung cancer mainly includes two types, one is non-small cell lung cancer (NSCLC), and the other is small cell lung cancer, with NSCLC accounting for over 85% of lung cancer [71]. NSCLC includes lung adenocarcinoma (LUAD), lung squamous cell carcinoma (LUSC), and large-cell carcinoma [72].

MiR-1-3p is downregulated in LUAD tissue, and upregulation of miR-1-3p levels demonstrates the ability to inhibit proliferation and migration in LUAD cells. In LUAD and adjacent tissues, there is a significant negative correlation between miR-1-3p and CENPF expression. CENPF expression is elevated in LUAD, with higher expression in the late stage (II + III + IV) compared to the early stage (I). There is a strong correlation between CENPF and poor prognosis of patients [73]. Therefore, the miR-1-3p/CENPF axis may have an important regulatory effect on LUAD and be a potential therapeutic target. Liu et al.'s study showed that family with sequence similarity 83 member A (FAM83A) is overexpressed in lung cancer cells and is associated with low survival rates in patients. The silencing of FAM83A can inhibit the proliferation, invasion and migration of lung cancer cells, which may be related to the inhibition of epidermal growth factor receptor (EGFR)/mitogen activated protein kinase (MAPK)/choline kinase α (CHKA) signal transduction and activation. The overexpression of FAM83A is believed to be related to the downregulation of miR-1-3p expression level. Overexpression of miR-1-3p can reduce the expression of FAM83A, thereby exerting potential anti-tumor effects [74].

Miao et al. found that overexpression of miR-1-3p could inhibit the proliferation, migration, and invasion ability of lung cancer cells, which was related to targeting cadherin EGF LAG seven-pass G-type receptor 3 (CELSR3) [75]. In exploring the regulatory function of lncRNA RMRP in NSCLC, it was found that RMRP achieved its cancer promoting effect by silencing miR-1-3p [29]. This also indicates once again that low expression of miR-1-3p is an important factor in promoting lung cancer progression, and increasing the expression level of miR-1-3p will be a potential targeted treatment approach. In addition, the miR-1-3p-PAICS axis has also been reported to be involved in the glycolysis and nucleotide metabolism of NSCLC cells, thereby affecting the progression of NSCLC [76].

### Bladder cancer

Bladder cancer (BLCA) is one of the ten most common cancers in the world [77–79]. At present, the occurrence of BLCA is believed to be highly correlated with smoking [80–82].

Low expression of miR-1-3p was observed in BLCA tissues and cells. Increasing the expression level of miR-1-3p in BLCA cells can inhibit cell proliferation, colony formation, migration and invasion, promote mitosis to stagnate in the G0/G1 phase, and increase the ratio of apoptosis. This process is related to miR-1-3p targeting C–C motif chemokine ligand 2 (CCL2) [83]. The role of CCL2 has also been extensively studied in BLCA. CCL2 is highly expressed in BLCA, and CCL2 staining results in BLCA cells and immune cells are considered as prognostic biomarkers for BLCA patients [84]. In the study of the mechanism of heat shock protein 47 (HSP47) promoting angiogenesis in BLCA, it was found that the induction of CCL2 and the activation of the ERK pathway were the causes of HSP47-induced angiogenesis [85]. In the functional study of lncRNA LNMAT1, it was found that it could promote BLCA related lymphangiogenesis and lymphatic metastasis. The mechanism is that LNMAT1 can recruit hnRNPL to the CCL2 promoter, leading to an increase in H3K4 trimethylation, thereby activating the expression of CCL2. The increased CCL2 is secreted into the tumor microenvironment, promoting the recruitment of tumor associated macrophages (TAM), and then promoting lymphatic metastasis through the secretion of vascular endothelial growth factor C (VEGF-C) [86]. In summary, the miR-1-3p/CCL2 axis is a highly promising therapeutic target in BLCA and deserves the focus of researchers.

Core 1 beta1,3-galactosyltransferase 1 (C1GALT1) has the function of regulating the O-glycosylation of tumor related proteins. Changes in the expression of C1GALT1 can lead to changes in the glycosylation of glycoproteins on the cell membrane, including mucins, growth factor receptors, adhesion molecules, etc. This change can cause a shift in the interaction between cell membrane surface molecules and ligands, ultimately affecting the biological behavior of tumor cells [87]. Tan et al. found that the expression of C1GALT1 and product T antigen was highly expressed in BLCA and promotes malignant behaviors such as proliferation, colony formation, migration, and invasion of BLCA cells. Mucin16 (MUC16) has been identified as a C1GALT1 target glycoprotein in BLCA, and its silencing inhibits the proliferation and migration ability of BLCA cells. With further research, it has been found that the role of C1GALT1 in BLCA was regulated by the cHP1BP3/miR-1-3p axis. Therefore, the cHP1BP3/miR-1-3p axis is a potential diagnostic marker and therapeutic target for BLCA [38].

Zhang et al. found that miR-1-3p could inhibit the proliferation, migration, and invasion of BLCA cells by targeting glutaminase (GLS) [88]. In addition to the enhanced glycolysis process, the enhancement of glutamine decomposition is also a characteristic of tumor cells, and GLS is a key enzyme in the glutamine decomposition process. GLS can decompose glutamine (Gln) into glutamic acid (Glu), and then Glu generates α-ketoglutarate (α-KG) under the action of glutamic acid transaminase 1 (GOT1) [89, 90]. The α-KG is an important intermediate product of the tricarboxylic acid (TCA) cycle, and the increase of α-KG can promote the TCA cycle to produce more energy, nucleotides, lipids, amino acids and other substances required by cells, which is conducive to cell growth and survival [91–93].

In addition, miR-1-3p can also inhibit the proliferation, migration, invasion ability of BLCA cells and promote their apoptosis by targeting the BDNF-TrkB signaling axis [94]. In summary, low expression of miR-1-3p in BLCA demonstrates a promoting effect on cancer development, and restoring or even overexpressing miR-1-3p levels is a potential therapeutic approach for BLCA.

### Liver cancer

Liver cancer is a deadly malignant tumor, and although treatment methods are constantly improving, the five-year survival rate of patients is still very low [95]. Liver cancer is divided into three categories: hepatocellular carcinoma (HCC), intrahepatic cholangiocarcinoma (ICC), and mixed cancer. HCC accounts for the vast majority of liver cancer (approximately 90%) [96]. The high-risk factors for HCC mainly include hepatitis B virus/hepatitis C virus (HBV/HCV) infection, long-term alcohol consumption, non-alcoholic fatty liver disease (NAFLD), type 2 diabetes, aflatoxin, liver cirrhosis, obesity, etc. [53, 97, 98].

MiR-1-3p is downregulated in HCC cells, and overexpression of miR-1-3p can inhibit the proliferation of HCC cells and promote their apoptosis, which is related to targeting sex-determining region Y-box 9 (SOX9) [99]. SOX9, as a transcript factor, has also been further studied in HCC. Research has found that SOX9 could directly bind to the promoter region to induce C-X-C motif chemokine 5 (CXCL5) expression, then activate signal transduction of PI3K-AKT and ERK1/2, ultimately promoting the proliferation and invasion of HCC cells. In addition, the SOX9/CXCL5 axis also facilitates the infiltration of macrophages and neutrophils in tumor tissue [100]. According to reports, SOX9 can bind to the promoter region and stimulate the expression of lncRNA-MKLN1-AS, thereby promoting the proliferation, invasion, and EMT process of HCC cells [101]. It is worth noting that the expression and stability of SOX9 are related to maintaining tumor stem cell characteristics [102].

Chen et al.’s study showed that miR-1-3p is low expressed in HCC tissues and cells, while overexpression of miR-1-3p can inhibit HCC cell proliferation, migration, and invasion, and induce cell cycle arrest and apoptosis. This is related to targeting origin recognition complex bundle 6 (ORC6) [103]. ORC6 is associated with the I-IV phase, overall survival (OS), and relapse-free survival (RFS) of HCC and plays a crucial role in the initiation of DNA replication, DNA metabolism, cell cycle and other processes [104, 105].

High vascularity is one of the important characteristics of HCC and plays an important role in tumor growth and metastasis. Anti-tumor angiogenesis is considered an effective treatment for advanced HCC [106, 107]. Some scholars have found that thymoquinone (TQ) could inhibit diethylnitrosamine (DEN) induced angiogenesis and metastasis of HCC, which may be related to upregulating the expression level of miR-1-3p [108]. This means that miR-1-3p may become a potential target for inhibiting angiogenesis in HCC and may provide promising treatment options for HCC patients.

In addition, in the research of Tang et al., it is also proved that miR-1-3p can inhibit the proliferation of HCC cells and promote apoptosis, and more HCC cells stay in G0/G1 phase. The LncRNA TUG1/miR-1-3p/IGF1 axis has also been proven to exist in HCC cells, but further research is needed on its effects on HCC cells [25].

### Prostate cancer

Prostate cancer (PCa) is a common malignant tumor in men. In the United States, prostate cancer has become the leading malignant tumor with the highest number of new cases and the second highest number of deaths [95]. The high-risk factors for prostate cancer mainly include age, genetics, dietary fat, obesity, androgen levels, and so on [109, 110].

The expression level of miR-1-3p is downregulated in prostate cancer tissues and cells, and is associated with poor prognosis in patients. MiR-1-3p can inhibit the proliferation and colony forming ability of PCa cells, decrease the expression levels of cyclin-dependent kinase 2 (CDK2) and cyclin-dependent kinase 4 (CDK4), and make more cells stay in the G0/1 phase. This indicates that miR-1-3p may affect cell proliferation by intervening in the cell cycle process. Further research has identified E2F transcription factor 5 (E2F5) and PFTAIRE protein kinase 1 (PFTK1) as targets for miR-1-3p to function [111]. E2F5 is an important member of the E2F family and has been reported to promote cell cycle progression and proliferation [112]. PFTK1 is a new member of the CDK family and has been reported to accelerate the G0/G1-S phase transition, thereby regulating cell cycle processes [113]. During the experiment, it was once again confirmed that E2F5 and PFTK1 have a promoting effect on proliferation and cell cycle in PCa. In addition, miR-1-3p can significantly inhibit tumor volume in PCa bearing nude mice, and reduced expression of E2F5 and PFTK1 was detected in tumor tissue [111].

Dai et al. found that silencing of lncRNA MALAT1 could inhibit the expression of coronin 1C (CORO1C) by reducing the adsorption of miR-1-3p. This process inhibits the migration, invasion, and EMT progression of PCa cells [26]. Guo et al.’s study showed that miR-1-3p is not only associated with promoting the proliferation and migration of PCa cells, but also with bone metastasis (BM) of Gleason 3+4 PCa. LIM and SH3 protein 1 (LASP1) have been identified as a target for miR-1-3p, which may be involved in activating Wnt signaling through interactions with β-catenin [114].

### Esophageal cancer

Esophageal cancer (EC) ranks seventh in the world in incidence rate and sixth in mortality and about 70% of cases occur in males [53]. EC is mainly divided into esophageal squamous cell carcinoma (ESCC) and esophageal adenocarcinoma (EAC). In developing countries, ESCC is the main type of EC, and its high-risk factors may be overheated food and beverages, smoking, alcohol abuse, dietary composition, etc. [53, 115]. In developed countries, EAC has become the main type of EC. The high-risk factors for EAC may be overweight, gastroesophageal reflux, etc. [48]. In the future, the proportion of EAC in EC worldwide will continue to increase, and overweight may become an increasingly important factor [116].

MiR-1-3p was detected to be downregulated in EC tissues and cells, indicating a close correlation with EC [27, 31, 117]. Quercetin is a Natural product, which can inhibit the proliferation, colony formation, invasion and promote apoptosis of EC cells. In the study of its mechanism, it was found that the activation of miR-1-3p/ transgelin2 (TAGLN2) axis in EC cells induced by quercetin was an important factor for its anti-tumor function [117]. In the study of the mechanism of silencing lncRNA LINC01518 against ESCC, it was found that the silencing of LINC01518 could upregulate miR-1-3p, thereby inhibiting the PIK3CA/Akt pathway [31]. In addition, it was also found that silencing lncRNA MALAT1 could inhibit the migration and invasion of EC cells by upregulating miR-1-3p. This may be related to miR-1-3p inhibiting the downstream CORO1C/ tropomyosin 3 (TPM3) axis [27].

### Oral squamous cell carcinoma

The incidence rate of oral cancer ranks eighth, and about 95% of oral cancer is oral squamous cell carcinoma (OSCC) [1, 118]. The main risk factors are smoking, drinking and oral human papilloma virus (HPV) infection, and the number of HPV related oral cancer cases is growing every year [1, 80]. However, in regions such as South Asia, East Asia, and the Pacific Island, one of the main risk factors is excessive chewing of betel nuts [119].

The expression of miR-1-3p was significantly downregulated in OSCC tissues and cells [120]. Overexpression of miR-1-3p can inhibit the proliferation, migration, and invasion of OSCC cells, block the transition from G0/G1 phase to S phase, and induce cell apoptosis, which is related to targeted silencing of dickkopf homolog 1 (DKK1) [120].

### Ovarian cancer

Ovarian cancer (OA) is one of the common gynecological malignancies. Icariin is the main active ingredient of Epimedium, which can inhibit the proliferation of OA cells, induce cell cycle arrest in the G1/S phase, and promote cell apoptosis. The mechanism is that icariin upregulates the expression level of miR-1-3p, thereby inhibiting the transduction of the TNKS2/Wnt/β-catenin signaling pathway [121]. Qu et al. showed that miR-1-3p was able to block cell cycle progression and inhibit proliferation, migration and invasion of OA cells by targeting c-Met [122]. Importantly, miR-1-3p can increase the sensitivity of OA cells to ferroptosis by targeting FZD7 [123]. This means that it may be possible to improve the efficacy of some anti-tumor drugs. For example, cisplatin induces not only apoptosis but also ferroptosis in tumor cells [124].

Although, there have been some studies showing that MiR-1-3p has the ability to resist OA. However, it has also been suggested that the inhibitory effect of miR-1-3p in OA is very limited [125]. This indicates that the signaling or effector cascade of miR-1 has been dysregulated in OA.

### Breast cancer

Breast cancer (BC) is the most common cancer in women worldwide, ranking second in the number of cancer deaths in women [1]. According to the expression of estrogen receptor (ER), progesterone receptor (PR) and human epidermal growth factor receptor 2 (HER2), BC can be divided into hormone receptor positive breast cancer (ER + or/and PR +), HER2 positive breast cancer (ER-, PR-, HER2 +) and triple negative breast cancer (ER −, PR −, HER2 −) [126].

Many studies have shown that miR-1-3p is expressed in low levels in BC tissues and cells [127]. For HR + BC, miR-1-3p can inhibit the proliferation, migration and invasion of MCF-7 and ZR-7530 BC cells, and promote their apoptosis, which may be related to targeting Bcl-2 [127]. Liu et al. showed that miR-1-3p inhibited MCF-7 cell proliferation and motility and promoted apoptosis, mainly by targeting K-Ras and lncRNA MALAT1 [128]. Meanwhile, miR-1-3p can increase the sensitivity of MCF-7 cells to cisplatin and paclitaxel [127]. For HER2 + BC, miR-1-3p can inhibit the malignant phenotype of SKBR3 cells, which is also related to targeting K-Ras and MALAT1 [128]. More importantly, compared to SKBR3 cells, the expression level of miR-1-3p in SKBR3-LR cells (lapatinib-resistant cell lines) is lower [128]. Restoration of miR-1-3p partially reverses resistance to lapatinib in SKBR3-LR cells [128]. For triple-negative BC, miR-1-3p was able to inhibit the proliferation of MDA-MB-231 cells, which was thought to be mainly caused by a significant increase in apoptosis rate [129]. Unsurprisingly, miR-1-3p was also inhibitory for migration and invasion. These phenotypic changes may be related to targeting the MEK/ERK pathway, but further validation is needed [129]. For potential clinical value, miR-1-3p was effective in increasing the sensitivity of MDA-MB-231 cells to cisplatin [129]. MiR-1-3p also plays an important role in regulating breast cancer stem cells. Wu et al. showed that miR-1-3p was able to target ecotropic viral integration site-1** (**EVI-1) to inhibit proliferation and EMT-related genes in BCSCs and promote apoptosis [130]. Interestingly, miR-1-3p was able to trigger mitochondrial damage and promote mitochondrial autophagy in BCSCs, which was associated with targeting mitochondrial inner membrane organizing system 1 (MINOS1), glycerol-3-phosphate dehydrogenase 2 (GPD2), and interacting with leucine-rich pentatricopeptide-repeat containing (LRPPRC) proteins [131]. However, this phenomenon did not occur in tumor non-stem cells [131]. This provides new insights into the role of MiR-1-3p for mitochondria.

In conclusion, miR-1-3p plays an important inhibitory role for different subtypes of BC as well as breast cancer stem cells. Moreover, it plays a positive role in improving drug sensitivity. Therefore, miR-1-3p may have a very promising clinical potential.

### Other cancer

Renal cell carcinoma (RCC) is a common malignant tumor of the urinary system, which originates from the epithelial system of the renal parenchyma urinary tubules and accounts for the vast majority of renal malignant tumors [132]. MiR-1-3p exhibits low expression in RCC tissues and cells, and is associated with clinical pathological parameters such as capsule, lymph node metastasis, and vascular invasion. MiR-1-3p can inhibit the EMT process of RCC cells and weaken the ability of migration and invasion, which is related to targeting and silencing Fibronectin 1. The same results were also obtained in RCC xenograft tumor mice [133]. In previous studies, it was reported that Fibronectin 1 has the ability to promote tumor cell migration and invasion [134]. Therefore, the miR-1-3p/Fibronectin 1 axis is a target worthy of attention for inhibiting the migration and invasion of RCC.

Osteosarcoma (OS) is a common malignant tumor of bone that occurs mostly in adolescents [135, 136]. miR-1-3p is lowly expressed in OS tissues and cells. Overexpression of miR-1-3p is able to inactivate the Wnt/β-catenin pathway by targeting cyclin-dependent kinase 14 (CDK14), thereby inhibiting cell proliferation and cell cycle progression while promoting apoptosis [137]. Cell cycle-dependent kinases (CDKs) are a class of key regulatory enzymes that drive cell cycle transitions and are considered to be critical targets for regulating cancer progression [138–140]. CDK14 is an important member of the CDK family. It has been reported that miR-330-3p, miR-139, miR-216a, miR-1182, and miR-223 can all inhibit OS development by targeting CDK14 [141–145]. This also reflects the importance of CDK14 in regulating OS. Wnt/β-catenin is a very classical signaling pathway that initiates the transcription of a series of downstream target genes (such as c-myc, cyclin D1, etc.) [146]. Its aberrant activation promotes the proliferation and survival of OS cells [146].

**Fig. 3:**
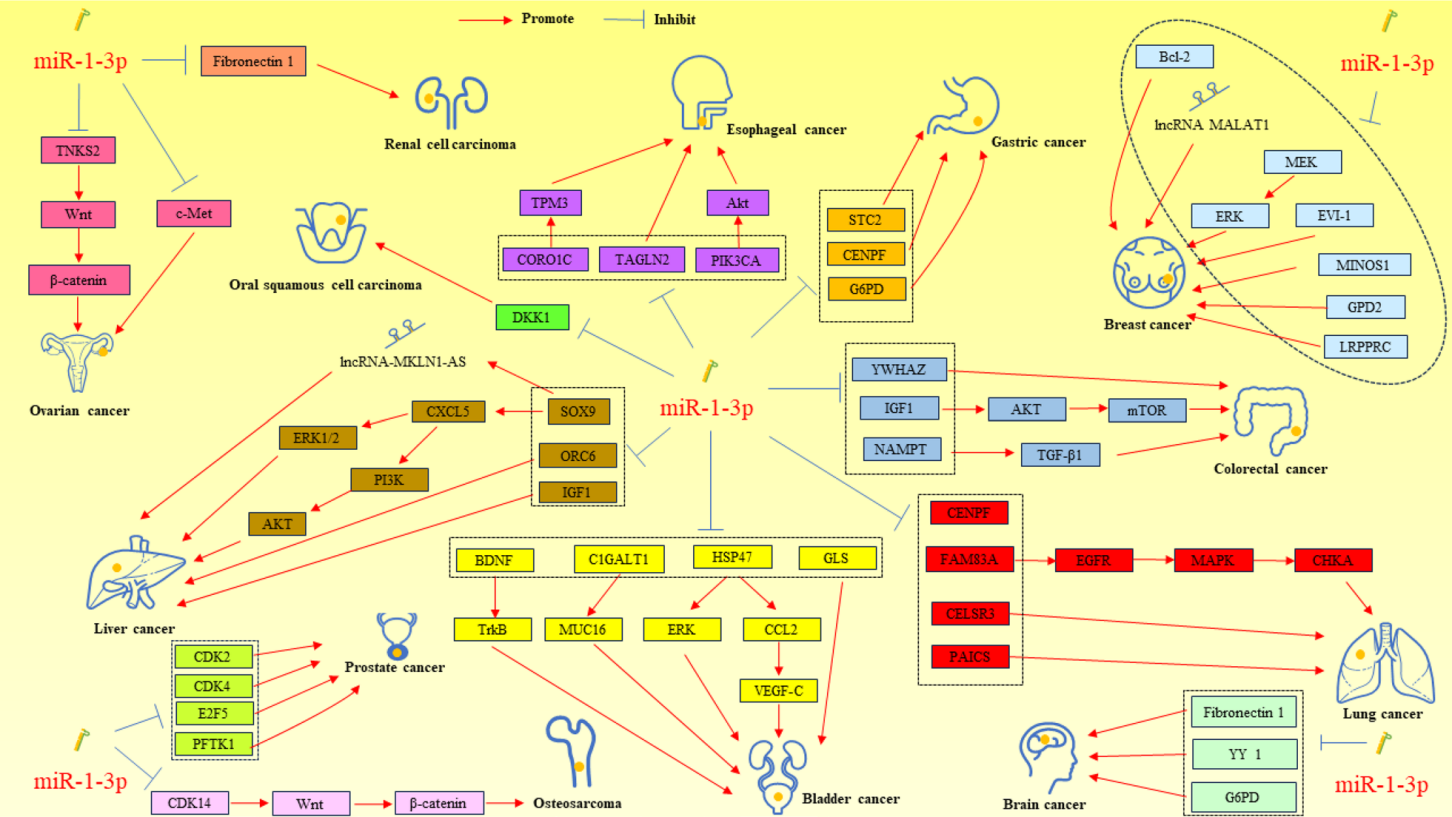
Role of miR-1-3p in tumor occurrence and development. Gastric cancer: STC2, stanniocalcin 2; CENPF, centromere protein F; G6PD, glucose-6-phosphate dehydrogenase. Colorectal cancer: YWHAZ, tyrosine 3/tryptophan 5 monooxygenase activation protein zeta; IGF1, insulin-like growth factor 1; mTOR, mammalian target of rapamycin; NAMPT, nicotinamide phosphoribosyl transferase; TGF-β1, transforming growth factor-β1. Lung cancer: CENPF, centromere protein F; FAM83A, family with sequence similarity 83 member A; EGFR, epidermal growth factor receptor; MAPK, mitogen activated protein kinase; CHKA, choline kinase α; PAICS, phosphoribosylaminoimidazole carboxylase. CELSR3, cadherin EGF LAG seven-pass G-type receptor 3; Bladder cancer: BDNF, brain-derived neurotrophic factor; TrkB, tyrosine kinase receptor B; C1GALT1, core 1 beta1,3-galactosyltransferase 1; MUC16, mucin16; HSP47, heat shock protein 47; ERK, extracellular regulated protein kinases; CCL2, C–C motif chemokine ligand 2; VEGF-C, vascular endothelial growth factor C; GLS, glutaminase; Liver cancer: SOX9, sex-determining region Y-box 9; CXCL5, C-X-C motif chemokine 5; PI3K, phosphatidylinositol-3-kinase; ERK1, extracellular regulated protein kinases 1; ERK2, extracellular regulated protein kinases 2; ORC6, origin recognition complex bundle 6; IGF1, insulin-like growth factor 1. Esophageal cancer: CORO1C, coronin 1C; TPM3, tropomyosin 3; TAGLN2, transgelin2; PIK3CA, phosphatidylinositol 4,5-bisphosphate 3-kinase catalytic subunit alpha isoform. Oral squamous cell carcinoma: DKK1, dickkopf homolog 1. Ovarian cancer: TNKS2, tankyrase 2; c-Met, cellular-mesenchymal epithelial transition factor. Breast cancer: Bcl-2, B-cell lymphoma 2; ERK, extracellular regulated protein kinases; EVI-1, ecotropic viral integration site-1; MINOS1, mitochondrial inner membrane organizing system 1; GPD2, glycerol-3-phosphate dehydrogenase 2; LRPPRC, leucine-rich pentatricopeptide-repeat containing. Other cancer: CDK14, cyclin-dependent kinase 14; YY1, Yin Yang 1; G6PD, glucose-6-phosphate dehydrogenase

miR-1-3p also plays an important regulatory role in brain tumors. For example, miR-1-3p was able to inhibit the proliferation and migration of glioblastoma (GBM) by targeting fibronectin and increase the sensitivity of GBM cells to temozolomide [147]. Zhang et al. found that lncRNA HOTAIR promoted the malignant phenotype of medulloblastoma, which was associated with targeting miR-1-3p/Yin Yang 1 (YY1) [148]. For pituitary tumors, miR-1-3p was able to inhibit NADPH production and glycolytic processes in pituitary tumor cells by targeting G6PD, causing inhibition of proliferation and promotion of apoptosis (Fig. 3) (Table 1) [149].

**Table 1:** Role of miR-1-3p in tumor occurrence and development

Target	Research object	Effect on tumor	References
STC2	Gastric cancer	Inhibit the proliferation and invasion	[56]
CENPF	Gastric cancer	Inhibit the proliferation, migration, and invasion	[57]
G6PD	Gastric cancer	Inhibit the Warburg effect Inhibit cell proliferation and promote cell apoptosis	[30]
YWHAZ	Colorectal cancer	Inhibit the proliferation and invasion	[65]
IGF1	Colorectal cancer	Inhibit the proliferation and promote their apoptosis	[66]
NAMPT	Colorectal cancer	Form a negative feedback pathway	[70]
CENPF	Lung adenocarcinoma	Inhibit proliferation and migration	[73]
FAM83A	Lung cancer	Inhibit the proliferation, invasion, and migration	[74]
CELSR3	Lung adenocarcinoma	Inhibit the proliferation, migration, and invasion	[75]
/	Non-small cell lung cancer	Inhibit the proliferation	[29]
PAICS	Non-small cell lung cancer	Involved in the glycolysis and nucleotide metabolism	[76]
CCL2	Bladder cancer	Inhibit cell proliferation, colony formation, migration, and invasion, promote mitosis to stagnate in the G0/G1 phase, and increase the ratio of apoptosis	[83]
C1GALT1	Bladder cancer	Inhibit the proliferation and migration	[38]
GLS	Bladder cancer	Inhibit the proliferation, migration, and invasion	[88]
BDNF/TrkB	Bladder cancer	Inhibit the proliferation, migration, invasion	[94]
SOX9	Hepatocellular carcinoma	Inhibit the proliferation of HCC cells and promote their apoptosis	[99]
ORC6	Hepatocellular carcinoma	Inhibit HCC cell proliferation, migration, and invasion, and induce cell cycle arrest and apoptosis	[103]
/	Hepatocellular carcinoma	Inhibit angiogenesis	[108]
IGF1	Hepatocellular carcinoma	Inhibit the proliferation of HCC cells and promote apoptosis, and more HCC cells stay in G0/G1 phase	[25]
E2F5 and PFTK1	Prostate cancer	Inhibit the proliferation and colony forming and make more cells stay in the G0/1 phase	[111]
CORO1C	Prostate cancer	Inhibit the migration, invasion, and EMT progression	[26]
LASP1	Prostate cancer	Inhibit the proliferation and migration and bone metastasis (BM) of Gleason 3 + 4 PCa	[114]
TAGLN2	Esophageal cancer	Inhibit the proliferation, colony formation, invasion and promote apoptosis	[117]
PIK3CA/AKT pathway	Esophageal cancer	Inhibit the proliferation and promote apoptosis	[31]
CORO1C/TPM3	Esophageal cancer	Inhibit the migration and invasion	[27]
DKK1	Oral squamous cell carcinoma	Inhibit the proliferation, migration, and invasion, block the transition from the G0/G1 phase to the S phase and induce cell apoptosis	[120]
Fibronectin 1	Renal cell carcinoma	Inhibit the EMT process and weaken the ability of migration and invasion	[133]
TNKS2/Wnt/ β-catenin signaling pathway	Ovarian cancer	Inhibit the proliferation, induce cell cycle arrest in the G1/S phase, and promote cell apoptosis	[121]
c-Met	Ovarian cancer	Block cell cycle progression and inhibit proliferation, migration, and invasion of OA cells	[122]
Bcl-2	Breast cancer	Inhibit proliferation, migration, invasion and promote cell apoptosis	[127]
K-Ras and lncRNA MALAT1	Breast cancer	Inhibited cell proliferation and motility and promoted apoptosis	[128]
MEK/ERK	Breast cancer	Inhibit proliferation, migration, invasion	[129]
BVI-1	Breast cancer	Inhibit proliferation and EMT-related genes in BCSCs and promote apoptosis	[130]
MINOS1, GPD2, LRPPRC	Breast cancer	Trigger mitochondrial damage and promote mitochondrial autophagy in BCSCs	[131]
CDK14	Osteosarcoma	Inhibit cell proliferation and cell cycle progression while promoting apoptosis	[137]
Fibronectin 1	Glioblastoma	inhibit the proliferation and migration of cells	[147]
YY1	Medulloblastoma	Promote malignant phenotypes	[148]
G6PD	Pituitary tumors	Inhibit NADPH production and glycolytic processes; Inhibit proliferation and promote apoptosis	[149]
VASP/Rap1 axis	Breast cancer	Inhibit the malignant phenotype and chemotherapy resistance of BC cells	[28]

In conclusion, miR-1-3p has been found to be down-regulated in a variety of tumors and closely associated with tumor development. Overexpression of miR-1-3p exhibited tumor suppression in a variety of tumor cells and animal models. Although, it is not clear whether these experimental results can be reproduced in the human body, this lays a preliminary theoretical foundation for clinical translation.

## Clinical significance of miR-1-3p

### Diagnostic marker

The study found that the expression level of miR-1-3p in serum was different between benign and malignant OA patients [150]. This seems to be helpful for the diagnosis of OA, but its diagnostic significance is lower than that of tumor marker C125 [150]. This limits the role of miR-1-3p in the diagnosis of OA. The research of Chen et al. shows that the expression level of miR-1-3p is significantly low in the serum of stomach adenocarcinoma (STAD) patients, and is closely related to the TNM stage and invasion depth of the patients. The level of miR-1-3p in serum has a certain degree of diagnostic ability. If combined with miR-125b-5p, miR-196a-5p, and miR-149-5p in serum, it can significantly improve the sensitivity and specificity of diagnosing STAD patients [151]. In addition, miR-1-3p in serum also plays an important role in the diagnosis of CRC [152]. Compared with the control group, the expression level of miR-1-3p in the serum of CRC patients was significantly reduced, and they had better predictive ability than carcinoembryonic antigen (CEA) and carcinoembryonic antigen 211 (CA211) [153]. In conclusion, the potential of miR-1-3p in tumor diagnosis still needs to be further developed. The combination of miR-1-3p and a variety of miRNAs may further improve the specificity and sensitivity of diagnosis, which will be conducive to clinical transformation.

### Prognostic marker

MiR-1-3p has good potential as a prognostic marker. The study found that patients with low serum miR-1-3p levels had higher all-cause mortality after abdominal aortic aneurysm (AAA) surgery [47]. Detecting the levels of miR-1-3p in patients before and after AAA surgery may help doctors determine the prognosis of AAA patients after surgery, in order to take appropriate intervention measures. The study of Wei et al. found that in patients who underwent radical prostatectomy for PCa, the expression level of miR-1-3p was significantly lower in the tumor tissues of the patients in the recurrence group compared with that in the no recurrence group. miR-1-3p was considered to be the only independent factor for prostate cancer recurrence [154]. This conclusion is similarly supported by the study of Karatas et al. [155]. Therefore, detecting the expression level of miR-1-3p in tumor tissues of PCa patients after radical prostatectomy for prostate cancer can help to provide physicians with information about the likelihood of the patient's cancer recurrence, so that relevant interventions can be prepared. In addition, NSCLC may lead to leptomeningeal metastases (LM), which is a terrible consequence. During the process of intrathecal chemotherapy for NSCLC-LM patients, the expression levels of miR-1-3p in the cerebrospinal fluid exosomes (CSF) of patients with partial response (PR) continuously increased compared to patients with progressive disease (PD) [156]. This suggests that miR-1-3p in CSF extracellular vesicles may become a biomarker for evaluating the efficacy of intrathecal chemotherapy in NSCLC-LM patients.

Currently, the study of miR-1-3p in the prognosis of tumors still needs a lot of exploration. In general, decreased expression levels of miR-1-3p are associated with poor prognosis, while the opposite is true for increased expression levels. However, long-term use of cardiotoxic drugs can also cause elevated serum levels of miR-1-3p, which requires special attention.

### Toxicity marker

Doxorubicin (DOX) is a common and potent anticancer drug, but its cytotoxicity is not specific. As a result, it also damages normal cells, such as myocardial cells, which is considered one of the main side effects of DOX [157]. MiR-1-3p was previously considered a specific miRNA in myocardial and skeletal muscles, which is released into the bloodstream during myocardial and skeletal muscle injuries. Rigaud et al. found that the increased expression of miR-1-3p in serum was closely related to cardiac dysfunction in BC patients who used DOX continuously. The ability of serum miR-1-3p to differentiate between patients with DOX-related myocardial injury and those without myocardial injury was superior to the common myocardial injury marker cardiac troponin I (cTnI) (Table 2) [158].

**Table 2:** Clinical significance of miR-1-3p

Function	Effect	Sample	Notes	References
Diagnostic marker	Distinguishing between benign and malignant OA	Plasma	The diagnostic significance is lower than that of tumor marker C125	[150]
Diagnostic marker	Diagnosing patients with STAD	Plasma	Combine with miR-125b-5p, miR-196a-5p, and miR-149-5p in serum	[151]
Diagnostic marker	Diagnosing patients with CRC	Plasma	The predictive ability is better than CEA and CA211	[152, 153]
Prognostic marker	Predicting the risk of postoperative all-cause mortality in AAA patients	Plasma	/	[47]
Prognostic marker	Predicting the risk of recurrence after radical resection in patients with Pca	Tissue	The only independent factor for prostate cancer recurrence	[154, 155]
Prognostic marker	Evaluating the efficacy of intrathecal chemotherapy in patients with NSCLC-LM	Cerebrospinal fluid	/	[156]
Toxicity marker	Evaluation of DOX-induced cardiotoxicity in the treatment of BC patients	Plasma	The ability to distinguish between patients with DOX-related myocardial injury and those without myocardial injury is superior to cTnI	[158]

## MiR-1-3p and drug resistance

The emergence of drug resistance in tumors involves complex mechanisms, including changes in the expression levels of non-coding RNAs (such as miRNAs) [159–161]. The change of miRNA expression contributes to tumor cell survival and resistance to chemotherapy by regulating a series of downstream genes related to proliferation, cell cycle, invasion, metastasis, DNA repair and programmed cell death.

Gefitinib is a tyrosine kinase inhibitor (TKI), which has good reactivity to advanced NSCLC patients with epidermal growth factor receptor (EGFR) mutations. Unfortunately, shortly after the use of drugs, the emergence of drug resistance led to treatment failure. It is reported that hepatocyte growth factor (HGF) is overexpressed in about 61% of patients with acquired drug resistance [162, 163]. HGF can reduce the expression of miR-1-3p in cells and induce EGFR mutant NSCLC cells to be resistant to gefitinib. Overexpression of miR-1-3p can target c-Met (HGF receptor), thereby inhibiting the AKT/ERK signaling pathway and EMT process, ultimately restoring the sensitivity of cells to gefitinib [164].

The abnormal activation of the PI3K/AKT/mTOR pathway can promote the proliferation of tumor cells and endow various malignant tumors, including acute myeloid leukemia (AML), with resistance to chemotherapy. The PI3K/mTOR dual inhibitor BEZ235 can inhibit the proliferation and migration of multidrug-resistant AML cell lines (HL-60/VCR and K562/ADR), and improve their sensitivity to VCR and ADR. The mechanism is that BEZ235 can upregulate miR-1-3p, and then silence BAG4, EDN1, and ABCB1 (key regulators of cell apoptosis, migration, and multidrug resistance), and ultimately sensitize multidrug-resistant AML cells [165].

Cisplatin is one of the most commonly used anticancer drugs in clinical practice and can be used as an adjuvant chemotherapy drug for malignant gliomas. LncRNA DANCR has been found to be associated with cisplatin sensitivity in malignant gliomas. DANCR upregulates AXL by targeting five miRNAs, including miR-1-3p, thereby activating the transduction of the PI3K/Akt/NF-κB signaling pathway, ultimately endowing malignant glioma cells with resistance to cisplatin [32].

Navitoclax is a powerful Bcl-2 protein family inhibitor with anti-tumor activity against various tumor cells. As previously mentioned, SND1 can bind and degrade specific miRNAs through the SN domain. Inhibiting SND1 can enhance the sensitivity of colon cancer cells to navitoclax by upregulating the levels of miR-1-3p [52].

**Fig. 4:**
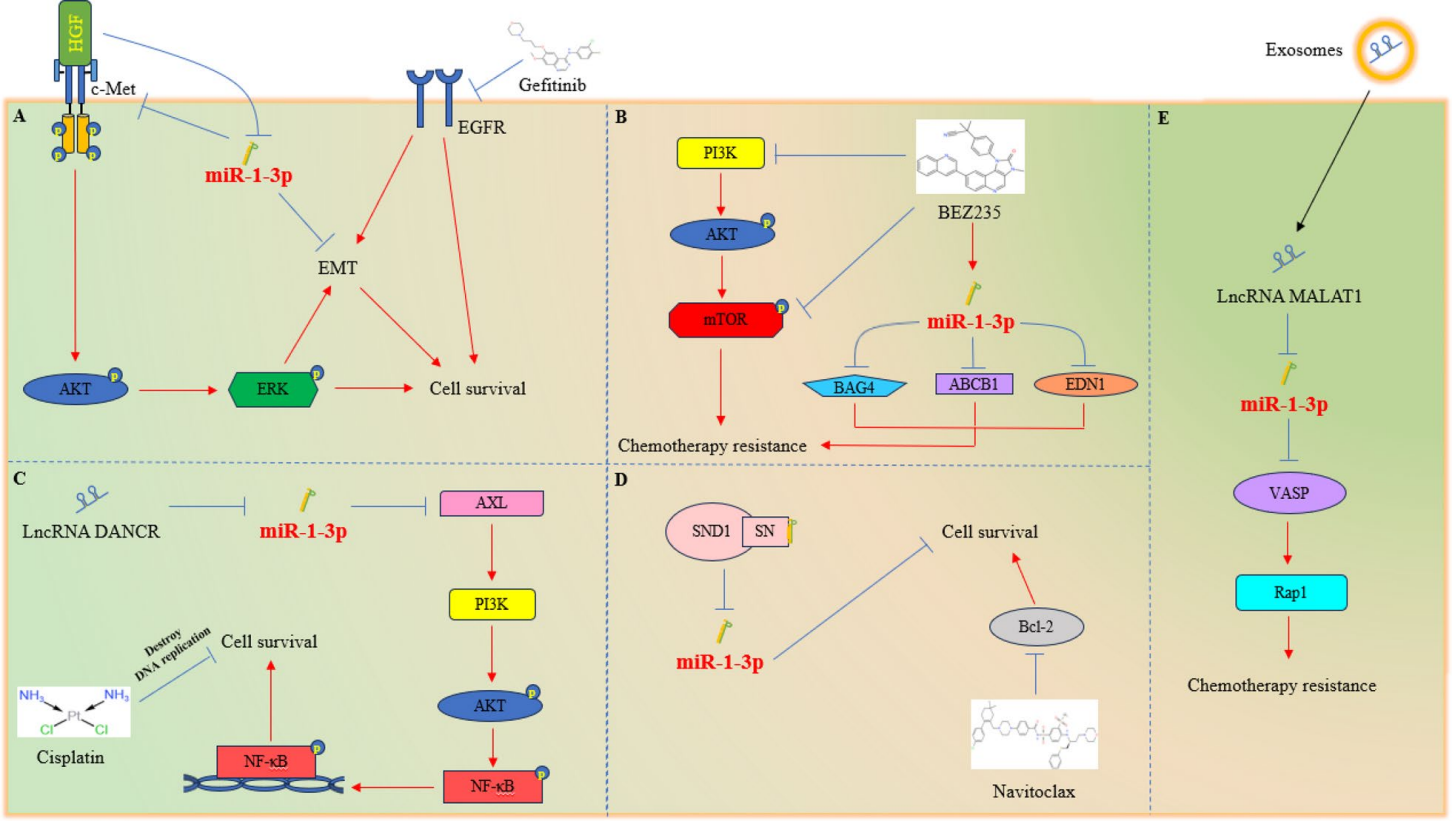
Role of miR-1-3p in chemotherapy sensitivity. **A** HGF can reduce the expression level of miR-1-3p and promote the resistance of NSCLC cells with EGFR sensitive mutations to Gefitinib. Overexpression of miR-1-3p can target c-Met, thereby inhibiting the AKT/ERK signaling pathway and EMT process, and ultimately restoring the sensitivity of NSCLC cells to Gefitinib. **B** The abnormal activation of the PI3K/AKT/mTOR signaling pathway endows AML cells with chemotherapy resistance. The dual inhibitor BEZ235 of PI3K and mTOR can upregulate the expression of miR-1-3p, thereby inhibiting BAG4, EDN1, and ABCB1, ultimately enhancing the chemotherapy sensitivity of AML cells. **C** LncRNA DANCR upregulates AXL by targeting five miRNAs, including miR-1-3p, thereby activating PI3K/Akt/NF- κB transduction of the signaling pathway, ultimately endowing GBM cells with resistance to cisplatin. **D** The SN domain of SND1 can bind and degrade miR-1-3p, giving colon cancer cells resistance to navitoclax. **E** BC cells endow surrounding BC cells with chemotherapy resistance through paracrine exosomes containing high expression levels of lncRNA MALAT1

In addition, the exosomes derived from BC cells transmit lncRNA MALAT1 to surrounding BC cells, which can silence miR-1-3p and activate the vasodilator-stimulated phosphoprotein (VASP)/RAS-associated protein 1 (Rap1) signaling axis, ultimately endowing BC cells with chemotherapy resistance (Fig. 4) [28].

## Approaches to targeting miR-1-3p

### Conventional drug-targeted methods

Conventional drugs including chemical drugs and natural drugs can target miR-1-3p and change its expression level. This is a very simple targeting method.

Propofol is a commonly used anesthetic in clinical settings. Ye et al. found that propofol could upregulate miR-1-3p in CRC cells, thereby inhibiting the activation of IGF1 and the AKT/mTOR axis, which was able to inhibit cell proliferation and promote apoptosis. Tumor growth in propofol-treated CRC xenograft nude mice was inhibited and upregulation of miR-1-3p could be detected, whereas silencing miR-1-3p reversed the efficacy of propofol [66]. However, propofol is strictly controlled due to its specific pharmacological effects and psychiatric dependence, which makes it difficult to be used as an agonist of miR-1-3p in the clinic. As research progresses, chemical agonists of miR-1-3p will continue to be discovered. Generally, chemical agonists are relatively inexpensive, but may also have more side effects.

Natural products have attracted the attention of many researchers because of their relatively low toxicity. Studies have found that quercetin could activate the miR-1-3p/TAGLN2 signaling axis in EC cells, thereby inhibiting cell proliferation and invasion and inducing apoptosis [117]. In addition, icariin was able to target the miR-1-3p/tankyrase 2 (TNKS2)/Wnt/β-catenin axis to inhibit the proliferation of OA cells and induce apoptosis [121]. Based on this theoretical basis, Fu et al. injected icariin into the peritoneum of OA xenograft nude mice, which effectively inhibited tumor growth. And in experiments, icariin was found to have less toxicity than cisplatin [121].

Although several chemical drugs and natural products are able to target miR-1-3p, they all share some common limitations. First, they have poor specificity and a rather large number of targets. Second, the mechanism of targeting miR-1-3p is also unclear [90]. Therefore, in order to better target the target molecule, gene drugs will become a hot spot for future research.

### Nano-delivery methods

MiR-1-3p expression is down-regulated in a variety of tumors, and delivery of miR-1-3p to tumor tissues is a promising gene therapy. However, miRNAs are negatively charged and not easily taken up by cells. In addition, they are very unstable in body fluids and are easily degraded by enzymes. Therefore, miRNA delivery is highly dependent on carriers. Vectors for the delivery of nucleic acids mainly include viral vectors and non-viral vectors. Viral vectors can effectively deliver miRNA into cells but are difficult to be further used for in vivo delivery due to factors such as biosafety risk, immune response, and small loading volume. Currently, non-viral vector delivery systems have become a research hotspot because of their diversity and modifiability. The same nanocarrier can deliver different miRNAs to target cells. Because there is currently little research on delivering miR-1-3p, the next step will be to introduce common miRNA delivery vectors and methods through other studies of miRNA delivery.

#### Lipid nanoparticle

Lipid nanoparticles (LNPs) are simple to prepare, have a large loading capacity, and are easy to produce on a large scale. LNP can increase their stability and targeting ability with some modifications.

Doxorubicin (DOX) is a broad-spectrum antitumor antibiotic for the treatment of HCC. However, its use is largely limited due to toxicity and chemotherapy resistance. miR-375 was able to inhibit the development of HCC by reducing the expression of Yes-associated protein 1 (YAP1), autophagy-related protein 7 (ATG7,) and astrocyte elevated gene-1 (AEG-1). It can also target multidrug resistance gene 1 (MDR1) and significantly inhibit DOX resistance. Fan et al. employed liposomal encapsulation of miR-375 and DOX to construct the L-miR-375/DOX NP complex. The complex was able to more effectively inhibit tumor growth in HCC-transplanted tumor-bearing mice and attenuated the cardiotoxicity and hepatotoxicity of DOX compared to DOX alone. In addition, the complex did not produce significant toxicity to the lungs, spleen, and kidneys. Xu et al. constructed a miR-101/DOX-L complex using liposome-encapsulated miR-101 and DOX, and used it for the treatment of HCC-transplanted tumor-bearing nude mice, and obtained similar results.

It has been reported that miR-603 expression was significantly reduced in GBM patients after radiotherapy, while the suppression of IGF1 and IGF1R expression was partially lifted, thus promoting cancer stem cell status and radiotherapy resistance [166]. Shabana et al. encapsulated the complex formed by miR-603 and polyethylenimine (PEI) with polyethylene glycol (PEG) and PR_b-modified liposomes. In this complex, PEG enhances the water solubility and biocompatibility of liposomes. PR_b is a fibronectin-mimetic peptide that can achieve targeting by specifically binding to integrin α5β1. PEI is a cationic polymer that helps miRNA escape from endosomes and lysosomes of cells. This complex can effectively elevate miR-603 in GBM cells and inhibit the expression of IGF1, thereby increasing the sensitivity to radiotherapy. This may be an effective strategy to improve radiotherapy resistance in GBM patients.

#### Metal nanoparticles

Inorganic metal nanoparticles are widely used in nucleic acid delivery studies, mainly including gold nanoparticles, superparamagnetic iron oxide nanoparticles (SPIONs), and mesoporous silica nanoparticles (MSNs).

Gold nanoparticles have unique optical properties, easy control of shape and size, good biocompatibility, and low cytotoxicity. Gold nanoparticles can be modified with PEI, PEG, lipoic acid, folic acid (FA), and other groups to enhance their encapsulation, biocompatibility, and targeting ability. Guo et al. constructed gold nanoparticles loaded with miR-21-3p (miR-21-3p-AuNp) and injected the complex into melanoma graft-tumor-bearing mice, which showed a significant increase in miR-21-3p in tumor tissues. MiR-21-3p was able to increase sensitivity to anti-PD-1 immunotherapy by promoting ferroptosis. The nanoparticles had low immunogenicity and did not significantly damage tissues such as heart, liver, spleen, lungs and kidneys, which demonstrated the high safety of gold nanoparticles [167]. In addition, gold nanoparticles have a strong near-infrared absorption capacity and can act as an anticancer photothermal agent in their own right. Huang et al. constructed anti-miR-181b/PTPAuNCs complexes using PEI-, PEG-, LA-, and FA-modified gold nanocages loaded with anti-miR-181b, a tumor suppressor. The complex was injected into HCC hormonal mice and irradiated the tumor site with near-infrared light, which was able to achieve the combination of gene therapy and photothermal therapy, thus significantly inhibiting tumor growth [168].

SPIONs are magnetically responsive nanoparticles that possess good biocompatibility, modifiability, low cytotoxicity, and degradability [169]. SPIONs can be directed to aggregate in tumor tissues under an applied magnetic field, and the magnetic field increases the nanoparticle's ability to penetrate the cell membrane and blood–brain barrier [170, 171]. It was reported that SPIONs loaded with miR-374a and SPIONs loaded with miR-326 were able to inhibit tumorigenicity in human glioma stem cell xenograft tumor mice and human endometrial cancer stem cell xenograft tumor mice, respectively [172, 173]. SPIONs can gather in the capillaries of tumor tissues under the action of the local magnetic field, thus blocking the blood supply of tumor tissues. For normal tissues in non-magnetic field areas, SPIONs are dispersed and do not block the blood vessels of normal tissues. In addition, SPIONS have magnetothermal effects and capabilities of magnetic resonance imaging (MRI) [174, 175]. After being localized in tumor tissues, SPIONs are able to gradually generate heat and warm up under the action of an alternating magnetic field, thus causing devastating damage to tumor cells [176]. This temperature controllability also enables the ability to achieve controlled drug release. However, this type of magnetothermal therapy is not suitable for combination with nucleic acid delivery, which may lead to degradation of the nucleic acid drug, and is therefore more suitable for combination with chemotherapeutic agents. As an excellent contrast agent, another property of SPIONs has the ability of MRI, which facilitates the diagnosis of cancer and visualization of nucleic acid delivery.

MSNs are solid nanoparticles with porous structure and large specific surface area. MSNs have good modifiability, biocompatibility, thermal stability, biodegradability, and are a good carrier for controlled release [177–179]. Garrido-Cano et al. constructed the MSN-PEI-miR200c-HA complex by wrapping MSNs with PEI to form a cationic surface to adsorb the negatively charged miR-200c-3p, and then wrapping a layer of hyaluronic acid (HA) around the outer layer. Among them, PEI mediated lysosomal escape and HA was able to bind CD44, which was highly expressed on BC cells, thus conferring targeting properties to the complex. Injection of the complex into BC xenograft tumor-bearing mice was able to accumulate in the tumor tissue and significantly increase the level of miR-200c-3p. miR-200c-3p was able to inhibit the expression of Zinc finger E-box binding homeobox 1 (ZEB1) and Zinc finger E-box binding homeobox 2 (ZEB2), which ultimately inhibited the ability of BC to grow [180].

#### Macromolecular polymer

PEIs, as mentioned previously, are cationic polymers with positively charged amino groups on their straight and branched chains capable of binding to negatively charged phosphate groups on miRNAs. Upon entering cancer cells, it is able to effectively escape from the lysosome, thus aggregating in the cytoplasm and releasing miRNAs [181]. However, PEI also has some limitations. Firstly, PEI is difficult to biodegrade in cells, and secondly, it is easy to combine with negatively charged proteins to produce cytotoxicity. Therefore, structural modification of PEIs is highly desirable. Zhang et al. constructed the R11-SSPEI/FAM-miR-145 complex using disulfide-bonded and polyarginine (R11)-modified PEIs loaded with FAM-tagged miR-145. The disulfide bond enhances the biocompatibility and degradability of PEIs, thereby reducing the toxic effects on cells.R11, a peptide that is specifically ingested in prostate cancer, confers targeting ability to PEIs. And FAM is a fluorescent dye that is capable of tracing the labeled nucleic acids. Injecting the complex into PCa-transplanted tumor-bearing mice was able to preferentially accumulate in tumor tissues, increase miR-145 levels, and effectively suppress tumors [182].

Chitosan can be obtained by partial deacetylation of chitin. Chitin is a natural polymer polysaccharide widely found in the shells of shrimps, crabs, insects and the cell walls of fungi, with good biocompatibility, biodegradability and non-toxicity [183]. However, the transfection efficiency of chitosan is relatively low, which can be improved by changing the molecular weight, degree of deacetylation, nitrogen-phosphorus ratio of chitosan, and by performing suitable chemical modifications [184]. Santos-Carballal et al. found that when chitosan had a molecular weight of approximately 40 kDa, a degree of acetylation of 12%, and a ( ±) charge ratio of 1.5, its transfection efficiency approached that of the harmaFECT and Novafect O 25 commercial reagents. Employing this chitosan complex loaded with miR-145 to transfect MCF-7 cells was able to significantly alter the levels of the corresponding target mRNAs without significant cytotoxicity [185].

There are over 200 types of dendritic macromolecules, including common ones such as polyamide amine (PAMAM), polylysine (PLL), and polypropylene imide (PPI). Dendritic macromolecules have a large cavity structure and a large number of positively charged amino groups, which are able to electrostatically bind to a large number of miRNAs, and have the advantages of large loading capacity, high transfection efficiency, good water solubility, and modifiability. Elfiky et al. constructed the LA-PAMAM/pmiR-218 complex using LA-modified hyperbranched PAMAM loaded with a plasmid expressing miR-128. The complex was able to effectively inhibit tumor development in HCC mice, where LA was able to specifically bind to the asialoglycoprotein receptor, which is highly expressed on HCC cells, thus conferring the complex targeting ability [186]. However, dendritic macromolecules also have some disadvantages, such as being difficult to biodegrade and having a large positive charge, which may cause some cytotoxicity if they accumulate in cells. Therefore, dendritic macromolecules modified with pegylation, glycosylation, acetylation, and peptide to neutralize some of the positive charges or to increase biodegradability may be effective measures to address the shortcomings.

Polylactic acid hydroxyacetic acid copolymer (PLGA) is a nano material with good biocompatibility and biodegradability. It can be decomposed into lactic acid and glycolic acid in the body and absorbed by the human body, so it has no cytotoxicity. PLGA is slowly degraded intracellularly and its degradation time is related to the ratio of lactic acid to hydroxyacetic acid, so it also has a controlled slow-release capability [187]. However, PLGA has a relatively low sample load and encapsulation rate [188, 189]. In addition, it is negatively charged under physiological conditions, which is unfavorable for cellular uptake. Wang et al. employed HA and PEI-modified PLGA to construct a HA-PEI-PLGA complex, which showed improved encapsulation rate and transfection efficiency. Treatment of triple-negative breast cancer cells MDA-MB-231 using the complex loaded with DOX and miR-542-3p significantly increased the content of both and promoted apoptosis [190].

In addition, many materials such as MOF and hydrogels can also be used as carriers for delivering miRNAs. In conclusion, all these vectors have their advantages and disadvantages (Table 3). In the future, more carriers will be developed and improved, which will provide technical support for miRNA delivery and clinical translation.

**Table 3:** Advantages and disadvantages of vectors

Type of vector	Advantages	Disadvantages
Viral vectors	High transfection efficiency	Biosafety risk, immune response, complex preparation, and small loading volume
Lipid nanoparticle	Simple preparation and good biocompatibility	Positively charged LNP may directly damage cell membranes and cause cytotoxicity
Gold nanoparticles	Easy control of shape and size, good biocompatibility, low cytotoxicity, and capability of photothermal effect	/
Superparamagnetic iron oxide nanoparticles	Good biocompatibility, modifiability, low cytotoxicity, degradability, Magnetic targeting, magneto-thermal effect, and magnetic resonance capability	/
Mesoporous silica nanoparticles	Good modifiability, biocompatibility, thermal stability, biodegradability	/
Polyethylenimine	High lysosomal escape ability and high transfection efficiency	Difficult to degrade and cytotoxic
Chitosan	Good biocompatibility, biodegradability and non-toxicity	Transfection efficiency is relatively low
Dendritic macromolecules	High transfection efficiency, good water solubility, and modifiability	Difficult to degrade and cytotoxic
Polylactic acid hydroxyacetic acid copolymer	Good biocompatibility biodegradability, and non-toxicity	A relatively low sample load, encapsulation rate, and transfection efficiency

## Prospects and conclusions

Cancer is a problem that mankind needs to address urgently, and although treatments have advanced over the years, its mortality rate remains high. This is mainly related to the complex mechanism of tumor development, the emergence of drug resistance, tumor recurrence, and other factors. Studying tumor development at the molecular level can help provide new ideas for cancer treatment. miRNAs are a class of non-coding RNAs that regulate a series of physiological processes by degrading mRNAs or inhibiting their translation through binding to the 3′-UTR of target mRNAs. miR-1-3p is an extremely important member of the miRNA family and was first found to be abundantly expressed in the cardiac and skeletal muscles and involved in their development. In recent years, miR-1-3p has been found to be significantly down-regulated in a variety of tumors and has an important role in tumor development, diagnosis, prognosis, and drug resistance, and is considered a tumor suppressor with great potential.

MiR-1-3p is encoded by the miR-1–2 gene located on chromosome 18q11.2 and is produced by shearing through a series of enzymes, which is similar to the production process of other miRNAs. Its level is regulated by a variety of factors, such as lncRNA, circRNA, DNA methylation, SNP, histone acetylation, and transcription factors. The study of the role of miR-1-3p in tumorigenesis and development is the theoretical basis for miRNA gene therapy. miR-1-3p expression levels are significantly down-regulated in a wide range of tumors, and its overexpression can effectively inhibit the malignant phenotype of tumors and promote their apoptosis. In terms of drug resistance, miR-1-3p can increase the sensitivity of some anti-tumor drugs. This may be related to its inhibition of cell survival-related signaling pathways, multidrug resistance genes, and reduction-related protein genes. In addition, miR-1-3p plays an important role in tumor diagnosis, prognosis, and drug toxicity assessment. The miR-1-3p in serum has good diagnostic potential in CRC, and its diagnostic value is superior to CEA and CA211. MiR-1-3p also has certain diagnostic capabilities in STAD, and its combined use with miR-125b-5p, miR-196a-5p, and miR-149-5p increases diagnostic accuracy. At present, there is limited research on the role of miR-1-3p in tumor diagnosis, and there is great research space. The combination of miR-1-3p with other miRNAs to construct a set of miRNA diagnostic panels and develop them into diagnostic kits is a promising direction. MiR-1-3p also plays an important indicator role in the prognosis of tumor patients, especially as the only independent factor for recurrence in patients after radical prostatectomy. The detection of miR-1-3p levels helps to understand the prognosis of patients so that relevant measures can be taken for intervention and improve their survival rate. In terms of drug toxicity assessment, due to the high expression of miR-1-3p in myocardial and skeletal muscles, when myocardial or skeletal muscle damage occurs due to drug use, intracellular miR-1-3p will be released into the bloodstream. Therefore, detecting the expression level of miR-1-3p in serum can evaluate the degree of drug-induced cardiotoxicity or skeletal muscle toxicity. It is not difficult to see that the long-term use of cardiotoxic or skeletal muscle toxic drugs will interfere with the diagnosis and prognosis of patients, so this point needs special attention.

At present, the clinical translation of miR-1-3p is full of opportunities and challenges, especially in targeted therapy. Currently, two siRNA gene drugs, patisiran and givosiran, have been approved by the FDA for clinical use, but miRNA drugs are still in the clinical trial stage. Among them, two studies are in Phase I, three studies are in Phase II, and five studies have been suspended or discontinued. For example, MRX34 (a mimetic of miR-34) was used in a clinical trial to treat melanoma, primary liver cancer, and hematological malignancies, but was forced to discontinue due to severe immune reactions in patients (NCT01829971). This may be related to the different functional characteristics of miRNA and siRNA. SiRNA is an exogenous RNA that binds to the translation region of mRNA and exerts silencing effects. The degree of sequence complementarity can reach 100%, and its target genes are generally 1–3. And miRNA is endogenous RNA that exerts silencing effects by binding to the untranslated region of mRNA, with a complementary degree of 20–90%, and its target genes ranging from dozens to even hundreds [191]. This means that there are too many targets for miRNA, which may lead to unknown side effects. Through functional enrichment analysis of the target genes of miR-34, it was found that there are two immune-related pathways (immune system and cytokine signaling in the immune system), including 28 approved drug target genes and 41 unapproved drug target genes [191]. Therefore, this provides a reasonable explanation for the serious immune-related adverse events caused by MRX34 treatment in the Phase I clinical trial.

**Fig. 5:**
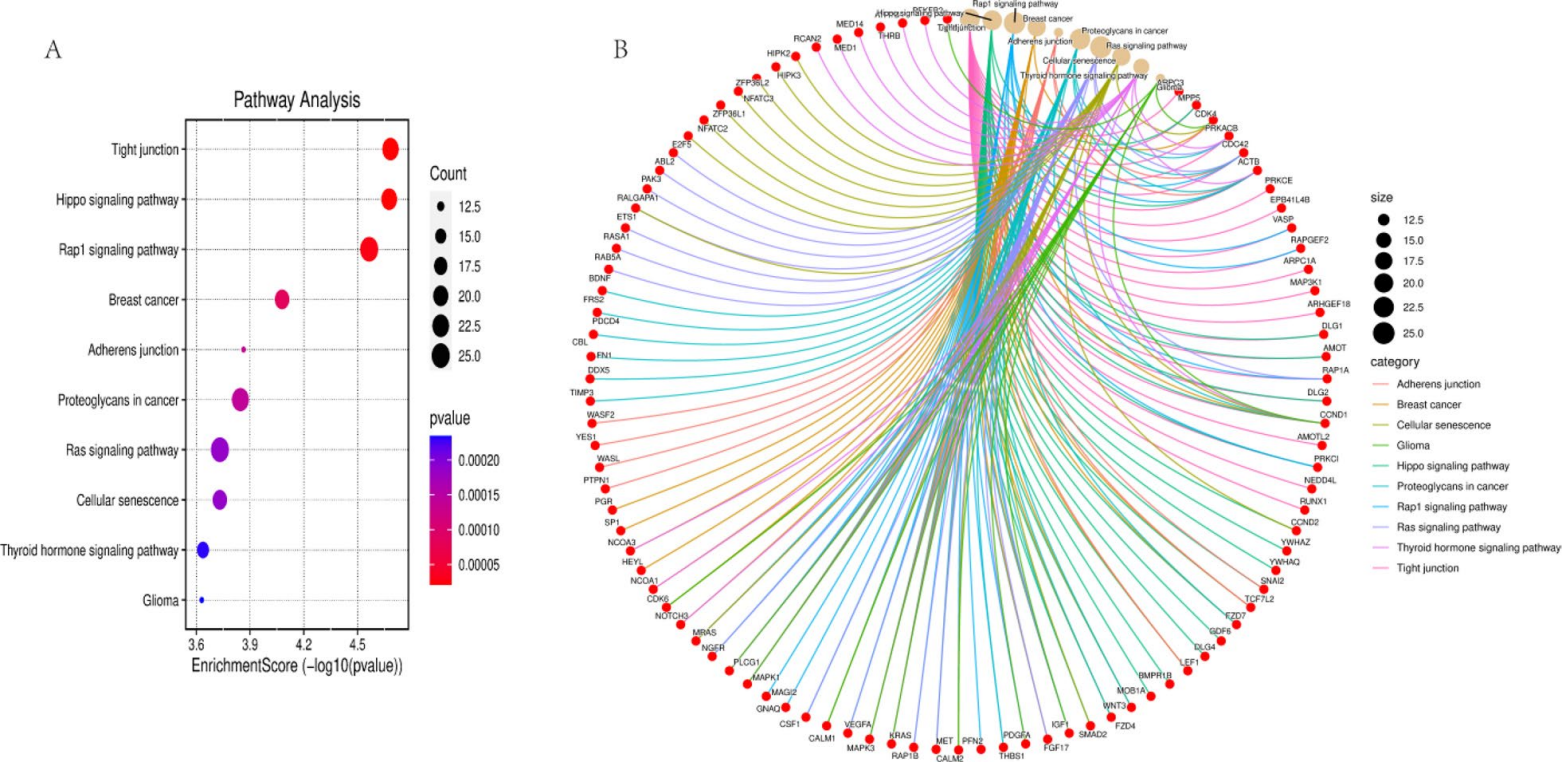
Pathway enrichment map of miR-1-3p targets. **A** The enrichment analysis of the miR-1-3p target pathway showed that the enrichment scores of tight junction, Hippo signaling pathway, Rap1 signaling pathway, breast cancer, adherens junction, proteoglycans in cancer, Ras signaling pathway, cellular senescence, thyroid hormone signaling pathway, and glioma ranked in the top ten. **B** Relevant targets in the pathways of the top ten enriched scores

MiR-1-3p similarly requires attention to the problems posed by multiple targets. Research has shown that overexpression of miR-1-3p increases the degree of injury in ischemia–reperfusion (I/R) mice, manifested as myocardial cell apoptosis and an increase in myocardial infarction area [192]. On the other hand, overexpression of miR-1-3p increases the risk of arrhythmia in normal or myocardial infarction rats [193]. In addition, in the diabetes rat model, high glucose induces the upregulation of miR-1-3p in cardiomyocytes through the MEK1/2 pathway and serum response factor (SRF) and then promotes cardiomyocyte apoptosis by targeting HSP60 [194]. Therefore, miR-1-3p has the potential to increase myocardial damage in patients with diabetes. Collect experimentally validated miR-1-3p targets through the TargetScan database (https://www.targetscan.org/vert_80/) for pathway enrichment analysis. The top 10 pathways with enrichment scores include tight junctions, Hippo signaling pathways, Rap1 signaling pathways, etc. (Fig. 5). These signaling pathways can become therapeutic targets in tumor cells, while they can also produce other different effects in other cells. According to reports, tight junction signaling pathways regulate cardiac conduction and intercellular communication, and Hippo and Rap1 signaling pathways are involved in the occurrence and development of I/R and arrhythmia [195–198]. This indicates that miR-1-3p plays an important role in cardiovascular disease, which is consistent with previous studies. If miR-1-3p enters other tissues, such as liver, spleen, lung, kidney, brain, etc., some unpredictable effects will occur. Therefore, too many targets are one of the main reasons for the slow development of miRNA drugs. With the rapid development of active targeting vectors, it brings new hope to the drug development of miRNA. Nanoparticles with active targeting ability can effectively deliver miRNAs to tumor sites, thereby avoiding or weakening its impact on other healthy tissues. With the continuous development of miRNA theory research and carrier research, miR-1-3p is likely to be able to achieve clinical translation in the near future, bringing benefits to cancer patients.

## Data Availability

Not applicable.

## Abbreviations

miRNA: MicroRNA
3′-UTR: 3′-Untranslated region
MyoD: Myogenic differentiation antigen
Mef2: Myocyte enhancer factor 2
Hand2: Heart and neural crest derivatives-expressed transcript 2
MIB1: Mindcomb homolog 1
pri-miRNA: Primary miRNA
pre-miRNA: Precursor miRNA
RISC: RNA-induced silencing complex
lncRNA: Long non-coding RNA
ceRNA: Competitive endogenous RNA
circRNA: Circular RNA
C1GALT1: Core 1 beta1,3-galactosyltransferase 1
GOLPH3: Golgiphosphoprotein 3
JUP: Junction plakoglobin
SNP: Single nucleotide polymorphism
AAA: Abdominal aortic aneurysm
SND1: Staphylococcal nuclease and tudor domain containing 1
TLR4: Toll like receptor 4
GC: Gastric cancer
STC2: Stanniocalcin 2
CENPF: Centromere protein F
G6PD: Glucose-6-phosphate dehydrogenase
PPP: Pentose phosphate pathway
NADPH: Nicotinamide adenine dinucleotide phosphate
GPX4: Glutathione peroxidase 4
GSH: Glutathione
FSP1: Ferroptosis suppressor protein 1
CoQH_2_: Reduced Coenzyme Q
GCH1: GTP cyclohydrolase 1
BH4: Tetrahydrobiopterin
DHODH: Dihydroorotate dehydrogenase
ROS: Reactive oxygen species
NOX: NADPH oxidase
CRC: Colorectal cancer
YWHAZ: Tyrosine 3/tryptophan 5 monooxygenase activation protein zeta
EMT: Epithelial mesenchymal transition
IGF1: Insulin-like growth factor 1
mTOR: Mammalian target of rapamycin
IGF1R: Insulin-like growth factor 1 receptor
TGF-β: Transforming growth factor-β
TGF-β1: Transforming growth factor-β1
NAMPT: Nicotinamide phosphoribosyl transferase
NSCLC: Non-small cell lung cancer
LUAD: Lung adenocarcinoma
LUSC: Lung squamous cell carcinoma
FAM83A: Family with sequence similarity 83 member A
MAPK: Mitogen activated protein kinase
CHKA: Choline kinase α
CELSR3: Cadherin EGF LAG seven-pass G-type receptor 3
PAICS: Phosphoribosylaminoimidazole carboxylase
BLCA: Bladder cancer
CCL2: C–C motif chemokine ligand 2
HSP47: Heat shock protein 47
ERK: Extracellular regulated protein kinases
H3K4: Histone H3 lysine 4
TAM: Tumor-associated macrophages
VEGF-C: Vascular endothelial growth factor C
C1GALT1: Core 1 beta1,3-galactosyltransferase 1
MUC16: Mucin16
GLS: Glutaminase
Gln: Glutamine
Glu: Glutamic acid
α-KG: α-Ketoglutarate
GOT1: Glutamic acid transaminase 1
TCA: The tricarboxylic acid
BDNF: Brain-derived neurotrophic factor
TrkB: Tyrosine kinase receptor B
HCC: Hepatocellular carcinoma
ICC: Intrahepatic cholangiocarcinoma
HBV: Hepatitis B virus
HCV: Hepatitis C virus
NAFLD: Non-alcoholic fatty liver disease
SOX9: Sex-determining region Y-box 9
CXCL5: C-X-C motif chemokine 5
PI3K: Phosphatidylinositol-3-kinase
ERK1: Extracellular regulated protein kinases 1
ERK2: Extracellular regulated protein kinases 2
ORC6: Origin recognition complex bundle 6
OS: Overall survival
RFS: Relapse-free survival
TQ: Thymoquinone
DEN: Diethylnitrosamine
PCa: Prostate cancer
CDK2: Cyclin-dependent kinase 2
CDK4: Cyclin-dependent kinase 4
E2F5: E2F transcription factor 5
PFTK1: PFTAIRE protein kinase 1
CDK: Cyclin-dependent kinase
CORO1C: Coronin 1C
LASP1: LIM and SH3 protein 1
EC: Esophageal cancer
ESCC: Esophageal squamous cell carcinoma
EAC: Esophageal adenocarcinoma
TAGLN2: Transgelin2
PIK3CA: Phosphatidylinositol 4,5-bisphosphate 3-kinase catalytic subunit alpha isoform
TPM3: Tropomyosin 3
OSCC: Oral squamous cell carcinoma
HPV: Human papilloma virus
DKK1: Dickkopf homolog 1
RCC: Renal cell carcinoma
OA: Ovarian cancer
BC: Breast cancer
VASP: Vasodilator-stimulated phosphoprotein
Rap1: RAS-associated protein 1
STAD: Stomach adenocarcinoma
CEA: Carcinoembryonic antigen
CA211: Carcinoembryonic antigen 211
EV: Extracellular vesicle
LM: Leptomeningeal metastases
CSF: Cerebrospinal fluid exosomes
PR: Partial response
PD: Progressive disease
TKI: Tyrosine kinase inhibitor
EGFR: Epidermal growth factor receptor
HGF: Hepatocyte growth factor
c-Met: Cellular-mesenchymal epithelial transition factor
AML: Acute myeloid leukemia
VCR: Vincristine
ADR: Adriamycin
BAG4: BAG family molecular chaperone regulator 4
EDN1: Endothelin 1
ABCB1: ATP-binding cassette sub-family B member 1
AXL: Axl receptor tyrosine kinase
NF-κB: Nuclear transcription factor-κB
Bcl-2: B-cell lymphoma 2
EVI-1: Ecotropic viral integration site-1
MINOS1: Mitochondrial inner membrane organizing system 1
GPD2: Glycerol-3-phosphate dehydrogenase 2
LRPPRC: Leucine-rich pentatricopeptide-repeat containing
CDK14: Cyclin-dependent kinase 4
YY1: Yin Yang 1
TNKS2: Tankyrase 2
LNP: Lipid nanoparticle
DOX: Doxorubicin
YAP1: Yes-associated protein 1
ATG7: Autophagy-related protein 7
AEG-1: Astrocyte elevated gene 1
MDR1: Multidrug resistance gene 1
PEI: Polyethylenimine
PEG: Polyethylene glycol
MSNs: Mesoporous silica nanoparticles
FA: Folic acid
MRI: Magnetic resonance imaging
HA: Hyaluronic acid
ZEB1: Zinc finger E-box binding homeobox 1
ZEB2: Zinc finger E-box binding homeobox 2
PAMAM: Polyamide amine
PLL: Polylysine
PPI: Polypropylene imide
PLGA: Polylactic acid hydroxyacetic acid copolymer
SRF: Serum response factor
